# The potential epidemiologic, clinical, and economic impact of requiring schools to offer Physical Education (PE) classes in Mexico City

**DOI:** 10.1371/journal.pone.0268118

**Published:** 2022-05-06

**Authors:** Marie C. Ferguson, Sarah M. Bartsch, Kelly J. O’Shea, Diana M. Thomas, Timothy H. Moran, Mario Solano Gonzales, Patrick T. Wedlock, Sindiso Nyathi, Matthew Morgan, Kevin L. Chin, Sheryl A. Scannell, Daniel L. Hertenstein, Molly Domino, Kushi Ranganath, Atif Adam, Katherine Tomaino Fraser, Adam Fraser, Bruce Y. Lee

**Affiliations:** 1 Public Health Informatics, Computational, and Operations Research (PHICOR), CUNY Graduate School of Public Health and Health Policy, New York City, NY, United States of America; 2 Center for Advanced Technology and Communication in Health (CATCH), CUNY Graduate School of Public Health and Health Policy, New York City, NY, United States of America; 3 Department of Mathematics, United States Military Academy West Point, West Point, NY, United States of America; 4 Department of Psychiatry and Behavioral Sciences, Johns Hopkins School of Medicine, Baltimore, MD, United States of America; 5 Laureus Sport for Good Foundation, New York City, NY, United States of America; Qatar University College of Education, QATAR

## Abstract

**Background:**

Many schools have been cutting physical education (PE) classes due to budget constraints, which raises the question of whether policymakers should require schools to offer PE classes. Evidence suggests that PE classes can help address rising physical inactivity and obesity prevalence. However, it would be helpful to determine if requiring PE is cost-effective.

**Methods:**

We developed an agent-based model of youth in Mexico City and the impact of all schools offering PE classes on changes in weight, weight-associated health conditions and the corresponding direct and indirect costs over their lifetime.

**Results:**

If schools offer PE without meeting guidelines and instead followed currently observed class length and time active during class, overweight and obesity prevalence decreased by 1.3% (95% CI: 1.0%-1.6%) and was cost-effective from the third-party payer and societal perspectives ($5,058 per disability-adjusted life year [DALY] averted and $5,786/DALY averted, respectively, assuming PE cost $50.3 million). When all schools offered PE classes meeting international guidelines for PE classes, overweight and obesity prevalence decreased by 3.9% (95% CI: 3.7%-4.3%) in the cohort at the end of five years compared to no PE. Long-term, this averted 3,183 and 1,081 obesity-related health conditions and deaths, respectively and averted ≥$31.5 million in direct medical costs and ≥$39.7 million in societal costs, assuming PE classes cost ≤$50.3 million over the five-year period. PE classes could cost up to $185.5 million and $89.9 million over the course of five years and still remain cost-effective and cost saving respectively, from the societal perspective.

**Conclusion:**

Requiring PE in all schools could be cost-effective when PE class costs, on average, up to $10,340 per school annually. Further, the amount of time students are active during class is a driver of PE classes’ value (e.g., it is cost saving when PE classes meet international guidelines) suggesting the need for specific recommendations.

## Introduction

Many schools have been cutting physical education (PE) classes due to time, space, and budget constraints, which raises the question of whether policymakers should require PE classes in schools. For example, in the United States, the number of states requiring PE in elementary schools dropped from 43 in 2012 to 39 in 2016, and the number of states requiring PE in junior high dropped from 41 in 2012 to 37 in 2016 [1]. But will such cost cutting measures end up costing society much more? After all, studies have shown that PE classes can increase children’s physical activity and improve short-term health outcomes such as weight [2–8].

Since policymakers and school officials have many competing priorities for funding, they could benefit from better understanding what the cost-effectiveness of mandating PE class in schools may be. Moreover, most of the existing studies have focused on the short-term health effects of PE classes and not the longer-term ones such as the impact on the risk of health conditions such as cardiovascular disease and cancer, which should be accounted for when making policy decisions with a broader societal perspective.

The Laureus Sport for Good Projects Deportes Para Compartir and Proyecto Cantera in Mexico City represented an opportunity to address this question. Because of their work on these projects, Laureus sought to identify the key drivers behind low physical activity levels in Mexico City (e.g., approximately 40% of youth do not meet the World Health Organization’s [WHO] physical activity (PA) guidelines [9]), which contribute to the upward trends in obesity prevalence among school age children[10]. In fact, in 2016 Mexico had the highest prevalence, globally, of overweight or obesity among youth (e.g., 33.2%) [11,12]. Further, it has been identified that less than half of schools in Mexico City offer PE classes [13], despite it being a required subject [14]. Therefore, one of the questions that emerged was whether Mexico City should begin mandating that schools offer regular PE classes. In order to answer this question, we developed a computational simulation model representing four districts in Mexico City to determine if requiring PE in schools would be cost-effective and if so, how much each school could invest in PE classes and still remain cost-effective. Different simulation runs helped identify what some of the major drivers of cost-effectiveness may be and how this value may change when considering how PE classes may affect children’s PA beyond their school time.

## Methods

### Model structure

**Fig 1:**
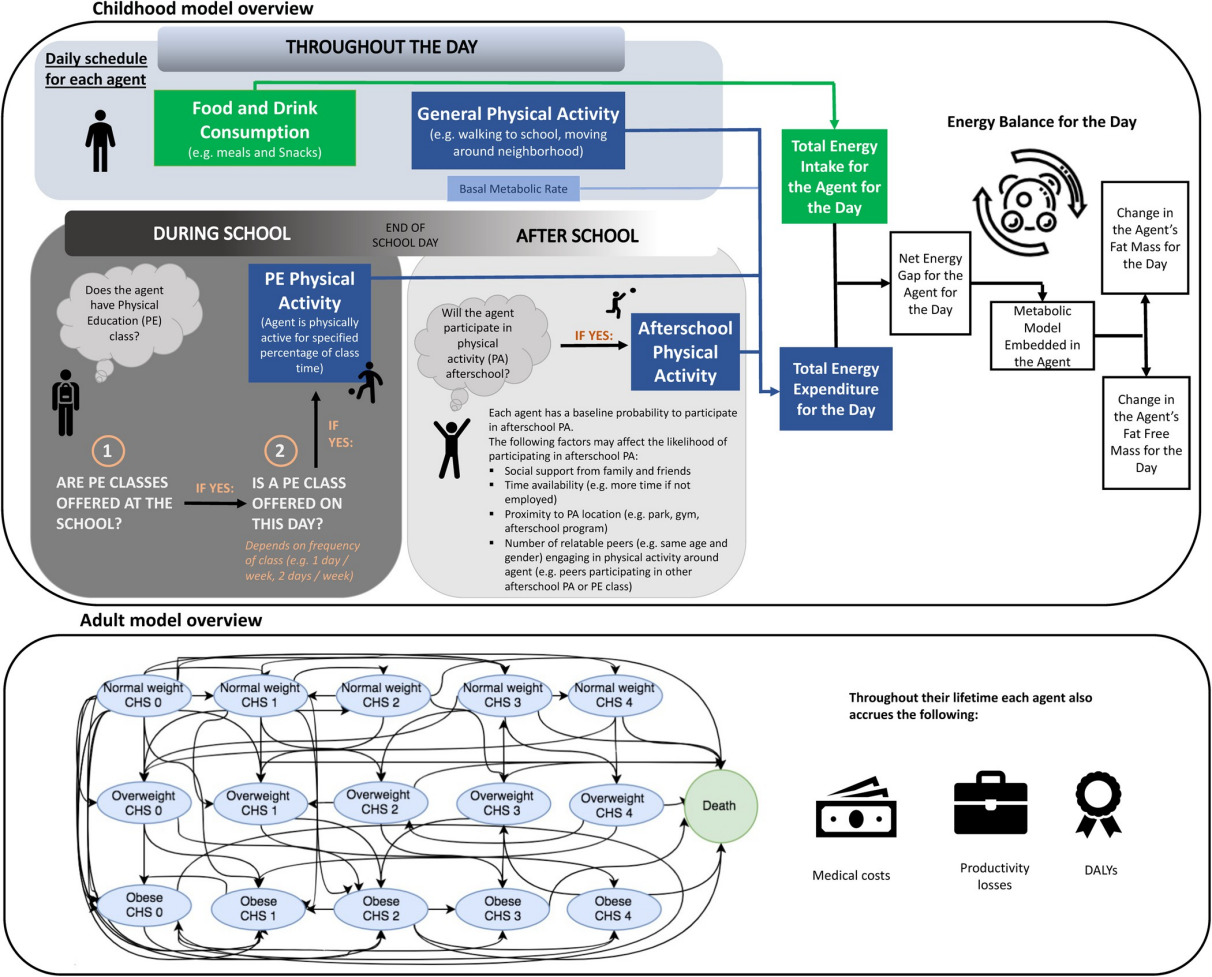
Model diagram.

Our Virtual Population for Obesity Prevention (VPOP) model is a geo-spatially explicit agent-based model written in the Python programming language [15–17]. We represented four districts in Mexico City (Benito Juárez, Cuauhtémoc, Miguel Hidalgo, and Coyoacán) as well as all homes, schools, and PA locations (parks and gyms). The model also includes a virtual representation of every school-age student between the ages of 6 and 18-years (total of 218,163 agents in the four districts, one for each student based on the actual student population in the four districts, according to Mexico’s 2010 census [18]). Each agent is assigned an age, sex, starting height and weight, and socioeconomic status, as well as a corresponding home and school location from a synthetic population to generate a human agent database (similar to that of the United States [19]). As shown in Fig 1, the model proceeds in one-day time steps for five years and simulates each agent going about their day, similar to real students in Mexico City, representing their diet, including food and drink consumption, as caloric intake. We determined the caloric intake for individuals based on obesity prevalence trends and then compared the caloric intake of agents (ranging up to approximately 2800 calories/day) to the typical caloric intake for children [20] and saw that it was similar. On school days, agents may or may not be able to participate in PE depending on whether or not the school offers it and if it is on the schedule for that day. If the agent’s school offers PE on a particular day, the agent would participate in activities for a proportion of each class, such as teams sports like soccer, basketball and volleyball, or running/jogging which correlates to moderate-to-vigorous PA, averaging 6 metabolic equivalents (METS) [21]. METS are the mass-specific rate of energy expenditure while participating in PA.

At the end of each school day, agents decide whether or not to participate in after school PA events, such as a team sport or unorganized play (see S1 Appendix). Each after school PA event (e.g., a single sports practice session or game) lasted 100 minutes [22], and requires an average intensity of 6 METS [21]. Agents consider various socio-cognitive and environmental factors (e.g., social support, finances, time, proximity to PA location) which affect their probability for participating in after school PA (S1 Appendix) [12,23–52] PE could also increase the likelihood of students being physically active outside of the PE class itself by increasing social norms to be physically active (e.g., if they see their peers active during class then they are more likely to participate in after school PA). On non-school days, agents do not have an opportunity to participate in PE but still make a decision about whether or not to participate in PA [53].

At the end of each day, PA results in the agent expending calories based on the intensity/duration of the PA and the agent’s current body weight. Agents maintained their caloric intake regardless of PA. As described in previous publications, an age- and sex-specific metabolic model embedded in each agent then converts caloric intake/expenditure into changes in fat mass, fat-free mass, and body mass index (BMI) [15,54,55].

When each agent reaches age 18 years old, the agent assumes one of 15 mutually exclusive states in a Markov model described in our previous publications [15,56] (See Supporting Information for descriptions of the different states.) Each simulated year, each student has state-, age-, and sex-specific probabilities of staying in the same state or transitioning to another state. While in a given health state, each simulated year, an individual has probabilities of developing various obesity-related health outcomes: stroke, coronary heart disease (CHD), type 2 diabetes, and cancers such as breast cancer, cervical cancer, colorectal cancer, esophageal cancer, kidney cancer, pancreatic cancer, stomach cancer, and uterine cancer for females, and colorectal cancer, esophageal cancer, kidney cancer, pancreatic cancer, prostate cancer, and stomach cancer for males. These probabilities draw from distributions based on the person’s underlying risk factors including sex and current age, health status, and BMI. Each simulated year, the person has an age-, sex-, and health state-specific probability of transitioning to the death state. These probabilities come from the WHO Global Health Observatory [57], while the probability of mortality associated with different health outcomes such as cancer, coronary heart disease (CHD) and strokes (S1 Appendix) come from the scientific literature.

### Economic measures

The third-party payer perspective includes direct medical costs (e.g., medications, outpatient visits, hospitalizations), while the societal perspective includes direct and indirect (i.e., productivity losses due to presenteeism) costs. Annual income attenuated by disability weights for an individual’s health condition served as a proxy for productivity losses. All individuals accrue productivity losses, regardless of age or employment status, as everyone is assumed to contribute to society.

For each scenario, we calculated the incremental cost-effectiveness ratio (ICER) as:

$$ICER=(Cost_{PE\;Classes}‐Cost_{No\;PE\;Classes})/(Health\;Effects_{No\;PE\;Classes}‐Health\;Effects_{PE\;Classes})$$

where health effects are measured in disability-adjusted life-years (DALYs). DALYs are the sum of the years of life lived with disability (YLD) and years of life lost (YLL) due to obesity outcome-related deaths. YLL and YLD are calculated as:

YLD=NumberofIncidentCases*DisabilityWeight*AverageDurationinYears

YLL=NumberofDeaths*LifeExpectancyatAgeofDeathinYears

If an individual had more than one health outcome, the outcome with the highest costs and health effect superseded the others (e.g., if an individual developed both stroke and CHD, the costs and disability weights associated with CHD would be accrued). All costs are reported in net present value (NPV) 2021 $US, converting all past and future costs using a 3% annual rate. Similarly, all future DALYs are presented in NPV, discounted with a 3% rate. We considered PE classes to be cost-effective if the ICER was less than or equal to Mexico’s gross domestic product (GPD) per capita (i.e., ≤$8,597 [58]) per DALY averted, following Mexico’s guide to evaluating health supplies [59], and economically dominant if it saved costs and provided health effects (e.g., cost saving).

### Data sources

S1 Appendix show the key model input parameters, values, and sources. All data came from the scientific literature or international databases. After school PA locations in Mexico City (e.g. parks, gyms, school grounds) were retrieved from Open Street Maps [60]. Currently observed PE class frequency and proportion of time students are physically active during PE class based on an observational study [13]. As previously described [15,56], state transition probabilities came from mid-sized and large longitudinal studies including (see S1 Appendix).

To convert pesos to $US, we first discounted costs to 2021 values using a 3% discount rate, then used the exchange rate (20.5 pesos/dollar, which was the average for 2021 as of November 12 [61]). In the absence of cost data specific to Mexico or a comparable country, we used the cost of treating the specific health conditions in the US as a proxy (S1 Appendix). To convert these costs into the cost of healthcare goods and services for these health conditions in Mexico, we first discounted them to 2021 values and then applied a ratio calculated using comparative price levels from the Organisation for Economic Co-operation and Development (OECD) for a representative basket of healthcare goods and services between the two countries [62]. In order to adjust for the difference in costs in years after diagnosis with cancer or end-stage renal disease (ESRD), we calculated the ratio of first year costs compared to subsequent year costs in the US and applied this ratio to the first year costs in Mexico. Annual income came from the reported national quarterly income which includes other benefits (e.g., fringe benefits, pensions) in addition to formal wages [63]. Disability weight values came for obesity-related outcomes came from the Global Burden of Disease 2019 [64].

### Experiments and sensitivity analyses

We evaluated the clinical and economic outcomes of implementing PE classes in Mexico City. Each experiment consisted of simulating agents’ (218,163 students ages 6 to 18 years) day to day PA, consumption, and metabolic outcomes for a duration of five years, then subsequent clinical and economic outcomes for the remainder of their lifetime (beginning at age 18). For the adult portion of the model, each simulation sent an 18-year-old through the Markov model and Monte Carlo simulations (i.e., probabilistic sensitivity analyses) consisting of 1000 trials simultaneously varying each parameter throughout their ranges (i.e., distributions, which account for variation and uncertainty in a value parameter across the population; S1 Appendix).

Our initial scenario assumes PE classes were not offered by any school in Mexico City, while experimental scenarios represented PE classes. Our initial PE scenario assumed that PE classes would occur once a week and last 40 minutes each class in primary schools and twice a week lasting 40 minutes each class in secondary schools. The scenario assumed for each class males would remain physical active during 32% of the class and females remain active for 26% of the class, based on qualitative and quantitative observations of PE classes in Mexico City [13,65]. The percent of time students remain active during the class is likely due to a combination of the curriculum, the instructor as well as the student’s motivation, self-efficacy, and self-perception. Subsequent experiments increase the amount of time students are active to represent changes to curriculum or their potential motivation or self-perception to participate or be active. The next PE scenario implemented PE classes that meet international PE recommendations (120 minutes of PE curriculum a week for primary schools and 180 minutes of PE curriculum a week for secondary schools, with students active for 50% of class) [66] in all schools. Sensitivity analyses varied the probability that students would choose to participate in after school activity based on whether they and their peers had participated in PE classes, and the cost of PE classes. We varied the cost of PE, total NPV over 5 years, from $42,032,248 to $50,333,172, and includes PE teacher wages and PE curriculum and equipment [67,68].

## Results

### Impact of requiring PE and not meeting class guidelines compared to no PE in schools

**Fig 2:**
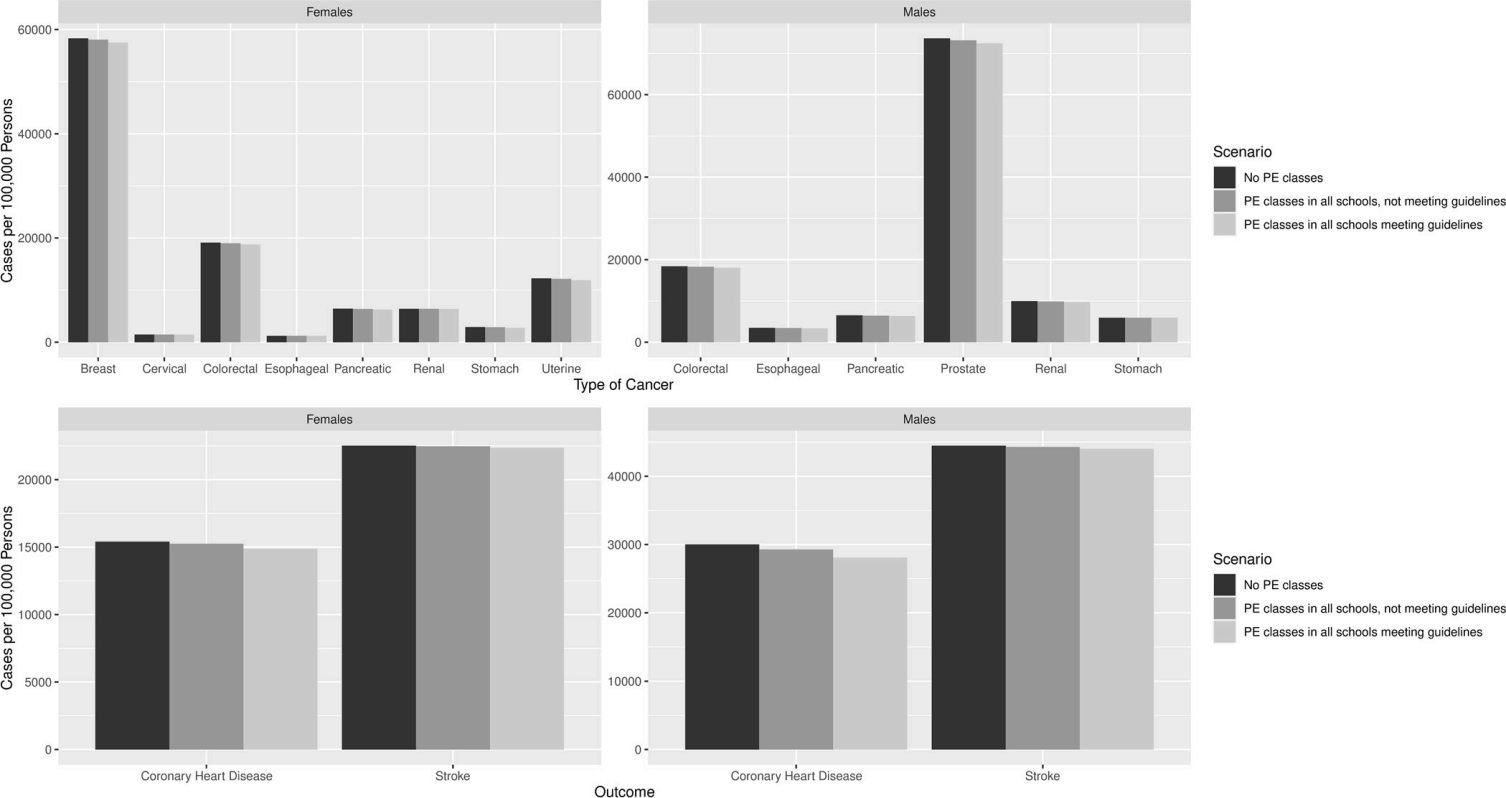
Cancer, stroke, and coronary heart disease (CHD) outcomes for males and females when no schools offer physical education (PE) classes and when requiring PE classes per 100,000 persons.

If all schools in Mexico City offered PE classes that did not meet international PE recommendations, overweight and obesity prevalence decreased by 1.5% (95% CI: 1.2%-1.8%) among males and 1.2% (95% CI: 0.76%-1.56%) among females compared to when there were no PE classes (overweight and obesity prevalence was 30.65% [95% CI: 30.58%-30.72%]). Across the lifetime of the population, PE classes averted 608 cases of cancer, 317 deaths due to cancer, 412 cases of CHD, 46 deaths due to heart attacks, and 99 strokes. Fig 2 shows the number of clinical outcomes, including different cancer types, with and without PE for males and females per 100,000 individuals. This allows us to compare how this early childhood intervention compares to other types of cancer prevention measures. When PE classes cost $42.0 million over the 5 years for all the schools (average $38,811 per school), this intervention was cost-effective from the third-party payer and societal perspectives, costing $3,595/DALY averted and $2,866/DALY averted, respectively. PE classes cost $4,744/DALY averted from the third-party payer perspective and $4,004/DALY averted from the societal perspective when costing $46.3 million over the 5 years. This increased to $5,786/DALY averted and $5,058/DALY averted (third-party payer and societal perspectives, respectively) when costing $50.3 million. PE classes could cost up to $56.1 million and remain cost-effective and up to $27.4 million and still provide cost savings (societal perspective).

Table 1 shows the total number of weight-related clinical outcomes, deaths and the corresponding economic outcomes for the simulated population.

**Table 1:** Clinical and economic outcomes when no schools offer physical education (PE) classes and when requiring PE classes.

	Weight-related health outcomes	Weight-related deaths	Disability-adjusted life years (DALYS)	Cost of PE classes	Direct medical costs due to weight-related outcomes	Productivity losses due to weight-related outcomes	Total societal costs
**No PE classes**	155,055	46,121	487,153.21	-	$4,010,214,129	$518,696,480	$4,528,910,608
**PE classes in all schools, not meeting guidelines**	153,936	45,750	476,034.37	50,333,172	$3,928,336,046	$510,527,231	$4,489,196,449
**PE classes in all schools, meeting guidelines**	151,871	45,039	483,366.01	50,333,172	$3,981,794,993	$515,937,633	$4,548,065,798
**PE classes affecting after school physical activity**	150,385	44,529	470,760.52	50,333,172	$3,889,928,195	$88,524,846	$4,446,926,447

### Impact of requiring PE and meeting class guidelines in all schools compared to no PE in schools

**Fig 3:**
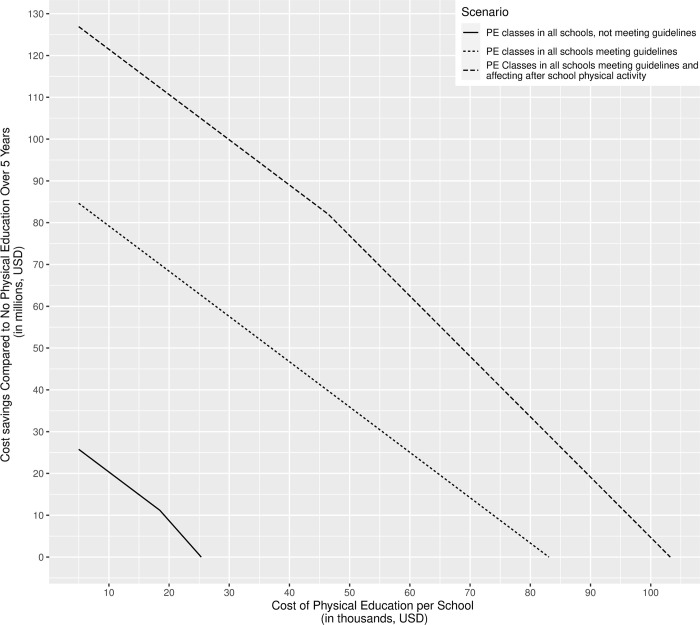
Societal cost savings from requiring physical education (PE) classes compared to no PE classes.

When all schools in Mexico City offered PE classes that met international recommendations for a PE curriculum, overweight and obesity prevalence decreased by 3.9% (95% CI: 3.7%-4.3%) among males and 4.0% (95% CI: 3.6%-4.4%) among females in the cohort at the end of 5 years. Long-term, this averted 1,788 cases of cancer and 941 deaths from cancer, 1,126 cases of CHD, 128 deaths due to heart attacks, and 270 strokes. However, there were 18 additional deaths from strokes among males due to individuals losing weight, living longer, and having more time, and a higher probability of developing negative health outcomes. When PE classes cost $42.0 million (total over 5 years; average $38,811 per school), they saved $39.9 million in direct medical costs and $48.0 million in societal costs, when they cost $50.3 million, they saved $31.5 million in direct medical and $39.7 million in societal costs. PE classes could cost up to $81.8 million over the 5 years and still be cost saving and up to $177.3 million to still be cost-effective from the third-party payer perspective (and up to $89.9 million and $185.5 million, respectively, from the societal perspective). Fig 3 shows the potential societal cost savings of PE class for different PE scenarios when varying the cost of implementing PE.

When PE classes influenced out-of-school PA, compared to no PE, overweight and obesity prevalence fell by 5.6% (95% CI: 5.3%-5.9%) among males and 6.1% (95% CI: 5.7%-6.5%) among females in the cohort at the end of 5 years. This averted 2,635 cancer cases, 1,389 deaths due to cancer, 1,639 cases of CHD, 187 deaths due to heart attacks, and 395 strokes across the simulated youth population in Mexico City, saving $69.9-$78.3 million in direct medical costs and $81.9-$90.3 million in societal costs, varying with the cost of PE classes ($42.0-$50.3 million). PE classes could cost up to $101.6 million over the 5 years and still provide cost savings and up to $2.9 billion and still remain cost-effective from the third-party payer perspective ($111.8 million and $2.9 billion, respectively, from the societal perspective).

## Discussion

Our study shows that requiring PE in all schools could be cost-effective when PE class costs, on average, up to $10,340 per school per year. This $10,340 figure would be higher than the cost of holding PE class in Mexico City, as it is higher than a PE teacher’s annual salary plus the cost of PE equipment. Our study shows that simply evaluating and accounting for the impact of PE on short-term outcomes, misses the downstream health benefits of PE classes that may take longer to manifest. To our knowledge, this is the first study demonstrating both the short and long-term health and economic benefits of requiring PE classes. Previous studies have primarily focused on the impact of PE class on increasing students’ total time physically active, as well as assessing if and how PE class affects students’ fitness (e.g., endurance, strength) [2–6]. Thus, our study adds to the current body of evidence to inform decision-making at the local, regional, and national level about how to invest in PE classes, given that there are many other competing priorities in schools.

Experimental scenarios revealed that a major driver of the cost-effectiveness of PE classes was the amount of time students remained active during each PE class. For example, when students are active for 50% of the class (e.g., schools met the international guidelines), PE classes would be cost saving (saving $39.7 million in societal costs). This showed that simply requiring PE classes may not be enough. After all, a study by Jennings-Aburto et. al of PE classes in Mexico City found that students may remain sedentary for more than 70% of a given PE class [13]. Therefore, our study suggests the need for specific guidelines regarding both the length of classes and amount of time students are active during the class. Further, strategies to monitor adherence need to accompany these guidelines to ensure students meet the recommended levels of PA during a given PE class.

Further, our study showed that PE classes could provide even more value when they resulted in students being more physically active outside of school. For example, accounting for PE’s potential influences on PA behaviors outside the classroom, could save an additional $42.3 million in societal costs and avert an additional 1,485 obesity-related health conditions. Thus, PE class curriculums may want to focus, for example, on demonstrating to students how to modify the activity or sport for participation at home or at a park, and teaching students to continue these activities outside of school. One example of this type of curriculum is the sports education model, which is a PE curriculum designed to develop students into “competent, literate, and enthusiastic players” by providing direct instruction, cooperative and peer learning which allows students to share the responsibility for things like practice strategies and their team’s success [69]. While research has demonstrated the short-term benefits of the sports education model on physical fitness and students’ motivation to participate in PA [70,71], to our knowledge, our study is the first to demonstrate that a program that can result in sustained PA engagement across the lifetime could lead to even greater health and economic benefits.

### Limitations

All models are simplifications of reality and cannot include all possible factors affecting the impact of PE class on direct and indirect medical costs. When calculating body weight changes, we assumed that participation in PE on a given day did not affect students energy intake (e.g., diet) or students participation in additional PA, as there is no evidence of a compensatory effect of PE class [72]. Our study focused on only four districts within the city, all of which are centrally located which may limit generalizability of our findings to other districts and populations. However, many conditions in our model are not just specific to Mexico City. For example, childhood overweight and obesity prevalence is comparable to other urban areas in the United States and other countries. Further, there are similar opportunities for PE and after school PA in school districts around the world and cuts to PE classes are occurring globally. Thus, our results and the principles of enhancing and expanding PE classes can be applied to other locations. While our model captures the health and economic benefits of PE, we did not account for other benefits of PE such as improved academic performance and improved social skills and emotional regulation.

### Conclusion

Our study shows that requiring PE in all schools could be cost-effective when PE class costs, on average, up to $10,340 per school per year. The amount of time students remained active during each PE class was a major driver of the cost-effectiveness (e.g., it is cost saving when PE classes meet international guidelines), suggesting the need for specific guidelines regarding the length of the classes and amount of time students are active, as well as strategies to monitor compliance. Further, our study showed that PE classes could provide even more value when they resulted in students being more physically active outside of school.

## Supporting information

S1 Appendix Supporting information for: The potential epidemiologic, clinical, and economic impact of requiring schools to offer Physical Education (PE) classes in Mexico City. (DOCX)
