# Supplementary material for: The potential epidemiologic, clinical, and economic impact of requiring schools to offer Physical Education (PE) classes in Mexico City
Source: PLoS One. 2022 May 6;17(5):e0268118. doi: 10.1371/journal.pone.0268118 (PMC9075653; doi:10.1371/journal.pone.0268118)
Supplement: S1 Appendix — (DOCX) [file pone.0268118.s001.docx]

**S1 Appendix.** Supporting Information for: The Potential Epidemiologic, Clinical, and Economic Impact of Requiring Schools to Offer Physical Education (PE) Classes in Mexico City.

*Decision to participate in after school physical activity*

The likelihood of participating in after school physical activity (PA) varied by agent. At the beginning of the simulation, each agent is categorized as a rare, sometimes, or usual after-school-exerciser, depending on their baseline probability to exercise based on the National Survey on Health and Nutrition (ENSANUT), which surveyed adolescents in Mexico to determine their participation in physical activity. This serves as a proxy to represent an individual’s past experiences and tendencies as well as individual-level drivers and barriers to exercise (e.g., presence or lack of motivation and self-perception, self-efficacy, abilities, etc.). Then various factors impact each agent’s initial baseline probability of participating in after school PA, resulting in a final probability of participating in after school PA. Support from friends and family members increases the probability of participating in after-school sports. Agents were randomly assigned as to whether or not they had supportive friends and family.[1] Financial barriers to PA were randomly assigned.[2] Sufficient time to participate depends on the student’s employment status[3] so that students who are employed have a lower probability to exercise.[3-5] Agents also consider how close other PA locations are relative to their school. Agents who have PA locations within 0.25 miles of their school have a higher probability to exercise, while agents who attend schools where the closest PA location is greater than 0.5 miles have a lower probability of participating in after school PA.[6]  Depending on the scenario, agents could also factor in the percentage of relatable agents who exercised the previous day (e.g., [(the number of agents who participated in PE and after school PA of the same gender and age)/(total agents of the same gender and age)]. If the percentage of relatable agents who exercised was higher than the average PA levels in Mexico City, than this functioned as a form of positive social pressure to participate.[7] We assumed a standard student could only participate in one after school PA event each day.

**Table S1**. Table of inputs for afterschool physical activity decision

|  | **Response Categories** | **Proportions** | **Source** |
| --- | --- | --- | --- |
| Base Preferences  Male, 10-13 years old | Usually Participate | 38.5 | [8] |
|  | Sometimes Participate | 24.4 |  |
|  | Rarely Participate | 37.1 |  |
| Base Preferences  Female, 10-13 years old | Usually Participate | 30 |  |
|  | Sometimes Participate | 27 |  |
|  | Rarely Participate | 43 |  |
| Base Preferences  Male, 14-16 years old | Usually Participate | 37.9 |  |
|  | Sometimes Participate | 26.7 |  |
|  | Rarely Participate | 35.4 |  |
| Base Preferences  Female, 14-16 years old | Usually Participate | 35.3 |  |
|  | Sometimes Participate | 24.9 |  |
|  | Rarely Participate | 39.8 |  |
| Base Preferences  Male, 17-18 years old | Usually Participate | 38.3 |  |
|  | Sometimes Participate | 25.1 |  |
|  | Rarely Participate | 36.6 |  |
| Base Preferences  Female, 17-18 years old | Usually Participate | 32.9 |  |
|  | Sometimes Participate | 24.6 |  |
|  | Rarely Participate | 42.5 |  |
| Social Group Influence - Male | Support from Family | 52.5 | [1] |
|  | No Support from Family | 47.5 |  |
|  | Support from Friends | 81.9 |  |
|  | No Support from Friends | 18.1 |  |
| Social Group Influence - Female | Support from Family | 52.6 |  |
|  | No Support from Family | 47.4 |  |
|  | Support from Friends | 64.2 |  |
|  | No Support from Friends | 35.8 |  |
| Time/Employment - Male, 6-13 years old | Yes employed (no time) | 6.5 | [3] |
|  | Not employed (yes time) | 93.5 |  |
| Time/Employment - Female, 6-13 years old | Yes employed (no time) | 3.3 |  |
|  | Not employed (yes time) | 96.7 |  |
| Time/Employment - Male, 14-17 years old | Yes employed (no time) | 32.3 |  |
|  | Not employed (yes time) | 67.7 |  |
| Time/Employment - Female, 14-17 years old | Yes employed (no time) | 15.1 |  |
|  | Not employed (yes time) | 84.9 |  |
| Proximity | High proximity | If location within 0.25 miles | [6] |
|  | Medium proximity | If location within 0.5 miles |  |
|  | No proximity | If no location within 0.5 miles |  |
| Percent of Relatable Agents who Participated in PE the Previous Day - Males | High | If >50% of peers participating in PE | [7] |
|  | Low | If <50% of peers participating in PE |  |
| Percent of Relatable Agents who Participated in PE the Previous Day - Females | High | If >50% of peers participating in PE |  |
|  | Low | If <50% of peers participating in PE |  |
| Density of Relatable Exercisers Afterschool Physical Activity - Males | High | If >50% of peers participating in PE | [7] |
|  | Low | If <50% of peers participating in afterschool physical activity |  |
| Density of Relatable Exercisers – Afterschool Physical Activity. - Females | High | If >50% of peers participating in afterschool physical activity |  |
|  | Low | If <50% of peers participating in PE |  |
| Perceived financial barrier | No barrier | 96.9 | [2] |
|  | Yes barrier | 3.1 |  |

*Chronic health states (CHS)*

The health states integrate the Edmonton Obesity Staging System (EOSS) and the Cardiometabolic Disease Staging System (CMDS).[9, 10] Each CHS is defined by six obesity-related comorbidity variables: fasting blood glucose, high-density lipoprotein (HDL), low-density lipoprotein (LDL), triglyceride, total cholesterol, and blood pressure.[11]

*Markov model transition probabilities*

State transition probabilities came from mid-sized and large longitudinal studies including Coronary Artery Disease Risk Development in Young Adults (CARDIA),[12] Atherosclerosis Risk in Communities (ARIC),[13] Framingham Heart Study (FRS),[14] Northern Manhattan Stroke cohort study,[15] Pittsburgh Epidemiology of Diabetes Complications Study,[16] and databases such as the National Institute of Cancer,[17] National Health Interview Survey,[18] and the US Renal Data System.[19]

**Table S2.** Markov model probability input parameters, values, and sources

| **Variables** | **Range** | **Parameters for which the range is across** | **Source** |
| --- | --- | --- | --- |
| **Probability of developing coronary heart disease (CHD)** | | | |
| at CHS2 | 0-0.04 | range across age, gender, and smoking status | [20, 21] |
| at CHS3 | 0-0.047 |  |  |
| at CHS4 | 0-0.063 |  |  |
| multiplier for increased probability due to being overweight | males: 1.22  females: 1.4 |  | [21] |
| multiplier for increased probability due to being obese | males: 1.58  females: 1.54 |  |  |
| **Probability of reoccurrence of CHD** | | | |
| at CHS2 | 0-0.1 | range across age, gender, and smoking status | [22] |
| at CHS3 | 0-0.057 |  |  |
| at CHS4 | 0-0.074 |  |  |
| **Probability of death due to CHD** | | | |
| at CHS2 | 0-0.04 | range across age, gender, and smoking status | [20, 21] |
| at CHS3 | 0-0.047 |  |  |
| at CHS4 | 0.0007-0.113 |  |  |
| multiplier for increased probability of death due to being overweight | males: 1.37  females: 0.98 |  |  |
| multiplier for increased probability of death due to being obese | males: 1.45  females: 1.3 |  |  |
| **Probability of developing Stroke** | | | |
| at CHS0 | 0-0.012 | range across age, gender, and smoking status | [20, 21, 23] |
| at CHS1 | 0-0.015 |  |  |
| at CHS2 | 0-0.017 |  |  |
| at CHS3 | 0-0.015 |  |  |
| at CHS4 | 0-0.028 |  |  |
| multiplier for increased probability due to being overweight | males: 1.17  females: 1.06 |  | [24, 25] |
| multiplier for increased probability due to being obese | males: 1.42  females: 1.23 |  |  |
| Probability of reoccurrence of Stroke at first year | males: 0.0924  females: 0.165 |  | [26] |
| Probability of reoccurrence of Stroke after first year | males: 0.0318  females: 0.0567 |  |  |
| **Probability of death due to stroke** | | | |
| In the first year of developing stroke | males: 0.135  females: 0.241 |  | [26] |
| After first year of developing stroke | males: 0.0596  females: 0.1064 |  |  |
| **Probabilities associated with diabetes complications** | | | |
| End stage renal disease | 0.0082 |  | [27] |
| Death from end stage renal disease | 0.081-0.239 | range across age and gender | [28] |
| Diabetic nephropathy | 0-0.28 | range across years of having type 2 diabetes mellitus | [16] |
| Diabetic neuropathy | 0-0.72 |  | [16] |
| Blindness | 0-0.8 |  | [29, 30] |
| **Probabilities of developing Cancer for females** | | | |
| Breast | 0-0.0191 | range across age | [17, 31] |
| Cervical | 0-0.0007 |  |  |
| Colorectal | 0-0.0066 |  |  |
| Esophageal | 0-0.0004 |  |  |
| Renal | 0-0.0018 |  |  |
| Pancreatic | 0-0.0025 |  |  |
| Stomach | 0-0.001 |  |  |
| Uterine | 0-0.0044 |  |  |
| **Probabilities of developing Cancer for males** | | | |
| Colorectal | 0-0.0071 | range across age | [17, 31] |
| Esophageal | 0-0.0013 |  |  |
| Renal | 0-0.0031 |  |  |
| Pancreatic | 0-0.0025 |  |  |
| Prostate | 0-0.0284 |  |  |
| Stomach | 0-0.0017 |  |  |
| **Probabilities of death due to cancer for females** | | | |
| Breast | 0.008-0.06 | range across age | [31] |
| Cervical | 0.006-0.083 |  |  |
| Colorectal | 0.027-0.105 |  |  |
| Esophageal | 0.035-0.426 |  |  |
| Renal | 0.011-0.057 |  |  |
| Pancreatic | 0.033-0.289 |  |  |
| Stomach | 0.03-0.314 |  |  |
| Uterine | 0.007-0.048 |  |  |
| **Probabilities of death due to cancer for males** | | | |
| Colorectal | 0.016-0.162 | range across age | [31] |
| Esophageal | 0.018-0.409 |  |  |
| Renal | 0.008-0.069 |  |  |
| Pancreatic | 0.018-0.335 |  |  |
| Prostate | 0.015-0.051 |  |  |
| Stomach | 0.015-0.264 |  |  |
| **Probabilities of death due to cancer for both genders** |  |  |  |
| multiplier for increased probability due to being overweight | males: 1.12  females: 1.14 |  | [32] |
| multiplier for increased probability due to being obese | males: 1.37  females: 1.41 |  |  |

**Table S3.** Markov model cost and disability weight input parameters, values, and sources

| **Variable** | **Distribution type** | **Mean (Range or standard deviation)** | | | **Source** |
| --- | --- | --- | --- | --- | --- |
| **Costs (2021 $US)** | | | | | |
|  |  | **Annual Costs** | | |  |
| Annual wages | Triangular | $8,227.75 ($7,404.98 – 9,050.53†) | | | [33] |
| CHS Stages |  |  | | |  |
| Stage 1 (CHS1) | - | $251.5 | | | [34]° |
| Stage 2 (CHS2) | - | $568.3 | | | [34]° |
| Normal weight stage 3 (CHS3) | - | $850.1 | | | [34]° |
| Overweight stage 3 (CHS3) | - | $1,039.9 | | | [34]° |
| Obese stage 3 (CHS3) | - | $1,988.7 | | | [34]° |
| Normal weight stage 4 (CHS4) | - | $1,470.5 | | | [34]° |
| Overweight stage 4 (CHS4) | - | $1,850.1 | | | [34]° |
| Obese stage 4 (CHS4) | - | $3,747.7 | | | [34]° |
| Diabetes mellitus 2 | Triangular | $4,420.90 ($3,978.81 – 4,862.99†) | | | [35] |
| Hypertension | Uniform | $99.64 – 365.4 | | | [36] |
| Diabetic nephropathy | Gamma | $300.1 ($111.3) | | | [37]° |
| Diabetic neuropathy | Gamma | $132.08 ($118.87 – 145.29†) | | | [38] |
| Diabetic retinopathy | Gamma | $271.60 ($244.44 – $298.76†) | | | [38] |
| Blindness | Gamma | $1,453.3 ($38) | | | [34]° |
| Stroke | Triangular | $5,404.05 ($4,863.64 – $5,944.45†) | | | [39] |
|  |  | **First Year** | **After First Year** | **Last Year** |  |
| Coronary heart disease |  |  |  |  |  |
| 18-44 years | Gamma | $10,086.7 ($12,561.7) | $2,620.2 ($7,425.0) |  | [34]° |
| 45-65 years | Gamma | $8,726.0 ($5,621.5) | $3,504.8 ($9,496.2) |  | [34]° |
| >65 years | Gamma | $6,944.8 ($4,718.2) | $2,074.7 ($5,857.8) |  | [34]° |
| End stage renal disease | Uniform | $23,283.29 – 33,573.44 | $15,316.11 – 22,085.13§ |  | [40] |
| Stomach cancer | Triangular | $17,624.21 ($15,861.79 - $19,386.63†) | $896.49 ($806.84 – 986.14†)§ | $29,236.26 ($26,312.63 - $32,159.88†)§ | [41] |
| Female cancer |  |  |  |  |  |
| Breast cancer | Gamma | $12,202.98 ($3.88) | $1,060.90 ($0.34)§ | $37,768.37 ($12.00)§ | [42, 43] |
| Cervical cancer | Triangular | $5,755.85  ($5,411.45 - $6,099.13‡) | $165.06  ($115.18 - $174.90‡)§ | $11,373.63 ($10,693.08 - $12,051.96‡)§ | [44] |
| Colorectal cancer | - | $28,570.6 | $1,598.6 | $53,461.8 | [45]° |
| Esophageal cancer | - | $44,270.7 | $3,467.8 | $65,960.3 | [45]° |
| Renal cancer | - | $21,373.3 | $3,165.2 | $46,708.8 | [45]° |
| Pancreatic cancer | - | $52,024.2 | $4,388.3 | $69,542.0 | [45]° |
| Uterine cancer | - | $14,903.7 | $776.8 | $44,388.7 | [45]° |
| Male cancers | - |  |  |  |  |
| Colorectal cancer | - | $12,846.1 | $1,116.8 | $39,759.0 | [45]° |
| Esophageal cancer | - | $28,570.6 | $721.1 | $49,688.3 | [45]° |
| Renal cancer | - | $28,570.6 | $1,598.6 | $53,461.8 | [45]° |
| Pancreatic cancer | - | $44,270.7 | $3,467.8 | $65,960.3 | [45]° |
| Prostate cancer | - | $21,373.3 | $3,165.2 | $46,708.8 | [45]° |
| **Disability Weights** | | | | | |
|  |  | **Annual** | | |  |
| Stroke | Triangular | 0.07 (0.046-0.099) | | | [46] |
| CHD | Triangular | 0.072 (0.047-0.103) | | |  |
| Diabetic nephropathy | Triangular | 0.052 (0.034-0.076) | | |  |
| Diabetic neuropathy | Triangular | 0.133 (0.089-0.187) | | |  |
| Diabetic retinopathy | Triangular | 0.031 (0.019-0.049) | | |  |
| End stage renal disease | Triangular | 0.571 (0.398-0.725) | | |  |
| Blindness | Triangular | 0.187 (0.124-0.26) | | |  |
| Hypertension | Triangular | 0.041 (0.026-0.062) | | |  |
| Type 2 diabetes | Triangular | 0.049 (0.031-0.072) | | |  |
|  |  | **First Year** | **After First Year** | **Last Year** |  |
| Breast cancer | Triangular | 0.288 (0.193-0.399) | 0.049 (0.031-0.072) | 0.54 (0.377-0.6887) |  |
| Colon cancer | Triangular | 0.288 (0.193-0.399) | 0.049 (0.031-0.072) | 0.54 (0.377-0.6887) |  |
| Esophageal cancer | Triangular | 0.288 (0.193-0.399) | 0.049 (0.031-0.072) | 0.54 (0.377-0.6887) |  |
| Uterine cancer | Triangular | 0.288 (0.193-0.399) | 0.049 (0.031-0.072) | 0.54 (0.377-0.6887) |  |
| Renal cancer | Triangular | 0.288 (0.193-0.399) | 0.049 (0.031-0.072) | 0.54 (0.377-0.6887) |  |
| Cervical cancer | Triangular | 0.288 (0.193-0.399) | 0.049 (0.031-0.072) | 0.54 (0.377-0.6887) |  |
| Pancreatic cancer | Triangular | 0.288 (0.193-0.399) | 0.049 (0.031-0.072) | 0.54 (0.377-0.6887) |  |
| Gastric cancer | Triangular | 0.288 (0.193-0.399) | 0.049 (0.031-0.072) | 0.54 (0.377-0.6887) |  |
| Prostate cancer | Triangular | 0.288 (0.193-0.399) | 0.049 (0.031-0.072) | 0.54 (0.377-0.6887) |  |

†Values are +/-10% of mean or median value

°US costs converted into the cost of healthcare goods and services for these health conditions in Mexico by applying a ratio calculated using comparative price levels from the Organization for Economic Co-operation and Development (OECD) for a representative basket of healthcare goods and services between the two countries

‡Values are 95% confidence interval

§Costs converted from first year costs in Mexico by applying the calculated ratio of first year costs compared to subsequent year costs in the US and applied this ratio to the first-year costs in Mexico

**References**

1. Fermino RC, Cassiano I, Rech R, Akira A, Hino F, Ciro I, et al. Physical activity and associated factors in high-school adolescents in Southern Brazil. Rev Saúde Pública. 2010;44.

2. Geografía INdEy. Módulo de práctica deportiva y ejercicio físico 2016. INEGI Mexico City; 2016.

3. ROSATI CF, RANZANI M, GUARCELLO L, LYON S, CAMPOS P. Experiencia mexicana en la reducción del trabajo infantil. Evidencia empírica y lecciones políticas R oma: U nderstanding C hildren’s W ork. 2012;11(4).

4. Work UCs, editor La experiencia mexicana en la reducción del trabajo infantil: evidencia empírica y lecciones políticas2012.

5. The Aspen Instute MUdVdM. Rediseño de la cultura física y el sistema deportivo mexicano para alcanzar su máximo potencial. <http://opinionpublicauvm.mx/es>: Universidad del Valle de Mexico, The Aspen Institute, 2016.

6. Yang Y, Diez-Roux AV. Walking distance by trip purpose and population subgroups. American journal of preventive medicine. 2012;43(1):11-9.

7. Bailey R, Wellard I, Dismore H, editors. Girls' Participation in Physical Activities and Sports: Benefits, Patterns, Influences and Ways Forward. Benefits of Physical Activity - Technical Papers of the WHO; 2004.

8. Morales-Ruan MDC, Hernandez-Prado B, Gomez-Acosta LM, Shamah-Levy T, Cuevas-Nasu L. Obesity, overweight, screen time and physical activity in Mexican adolescents. Salud publica de Mexico. 2009;51 Suppl 4:S613-20. doi: 10.1590/S0036-36342009001000016. PubMed PMID: 20464237.

9. Sharma AM, Kushner RF. A proposed clinical staging system for obesity. International journal of obesity. 2009;33(3):289-95.

10. Guo F, Moellering DR, Garvey WT. The progression of cardiometabolic disease: validation of a new cardiometabolic disease staging system applicable to obesity. Obesity. 2014;22(1):110-8.

11. Fallah‐Fini S, Adam A, Cheskin LJ, Bartsch SM, Lee BY. The additional costs and health effects of a patient having overweight or obesity: a computational model. Obesity. 2017;25(10):1809-15.

12. Friedman GD, Cutter GR, Donahue RP, Hughes GH, Hulley SB, Jacobs Jr DR, et al. CARDIA: study design, recruitment, and some characteristics of the examined subjects. Journal of clinical epidemiology. 1988;41(11):1105-16.

13. Investigators A. The atherosclerosis risk in communit (aric) stui) y: design and objectwes. American journal of epidemiology. 1989;129(4):687-702.

14. D'Agostino RB, Grundy S, Sullivan LM, Wilson P, Group CRP. Validation of the Framingham coronary heart disease prediction scores: results of a multiple ethnic groups investigation. Jama. 2001;286(2):180-7.

15. Sacco RL, Shi T, Zamanillo M, Kargman D. Predictors of mortality and recurrence after hospitalized cerebral infarction in an urban community: the Northern Manhattan Stroke Study. Neurology. 1994;44(4):626-.

16. Orchard TJ, Dorman JS, Maser RE, Becker DJ, Drash AL, Ellis D, et al. Prevalence of complications in IDDM by sex and duration: Pittsburgh Epidemiology of Diabetes Complications Study II. Diabetes. 1990;39(9):1116-24.

17. Fay MP, Pfeiffer R, Cronin KA, Le C, Feuer EJ. Age‐conditional probabilities of developing cancer. Statistics in medicine. 2003;22(11):1837-48.

18. Wolf AM, Colditz GA. Current estimates of the economic cost of obesity in the United States. Obesity research. 1998;6(2):97-106.

19. Saran R, Li Y, Robinson B, Ayanian J, Balkrishnan R, Bragg-Gresham J, et al. US Renal Data System 2014 Annual Data Report: Epidemiology of Kidney Disease in the United States. Am J Kidney Dis. 2015;66(1 Suppl 1):Svii, S1-305. Epub 2015/06/27. doi: 10.1053/j.ajkd.2015.05.001. PubMed PMID: 26111994; PubMed Central PMCID: PMCPMC6643986.

20. D'Agostino Sr RB, Grundy S, Sullivan LM, Wilson P. Validation of the Framingham coronary heart disease prediction scores: results of a multiple ethnic groups investigation. JAMA : the journal of the American Medical Association. 2001;286(2):180-7.

21. Wilson PW, D'Agostino RB, Sullivan L, Parise H, Kannel WB. Overweight and obesity as determinants of cardiovascular risk: the Framingham experience. Archives of internal medicine. 2002;162(16):1867-72.

22. Rea TD, Heckbert SR, Kaplan RC, Smith NL, Lemaitre RN, Psaty BM. Smoking status and risk for recurrent coronary events after myocardial infarction. Annals of Internal Medicine. 2002;137(6):494-500.

23. Pender JR, Pories WJ. Epidemiology of obesity in the United States. Gastroenterology Clinics of North America. 2005;34(1):1-7.

24. Rexrode KM, Hennekens CH, Willett WC, Colditz GA, Stampfer MJ, Rich-Edwards JW, et al. A prospective study of body mass index, weight change, and risk of stroke in women. Jama. 1997;277(19):1539-45.

25. Walker SP, Rimm EB, Ascherio A, Kawachi I, Stampfer MJ, Willett WC. Body size and fat distribution as predictors of stroke among US men. American Journal of Epidemiology. 1996;144(12):1143-50.

26. Sacco RL, Shi T, Zamanillo M, Kargman D. Predictors of mortality and recurrence after hospitalized cerebral infarction in an urban community The Northern Manhattan Stroke Study. Neurology. 1994;44(4):626-.

27. Kiberd BA, Clase CM. Cumulative risk for developing end-stage renal disease in the US population. Journal of the American Society of Nephrology. 2002;13(6):1635-44.

28. Saran R, Li Y, Robinson B, Abbott KC, Agodoa L, Ayanian J, et al. US Renal Data System 2015 Annual Data Report: Epidemiology of Kidney Disease in the United States. American journal of kidney diseases: the official journal of the National Kidney Foundation. 2016;67(3 Suppl 1):A7.

29. Yau JW, Rogers SL, Kawasaki R, Lamoureux EL, Kowalski JW, Bek T, et al. Global prevalence and major risk factors of diabetic retinopathy. Diabetes care. 2012;35(3):556-64.

30. Klein R, Klein BE, Moss SE, Cruickshanks KJ. Relationship of hyperglycemia to the long-term incidence and progression of diabetic retinopathy. Archives of internal medicine. 1994;154(19):2169-78.

31. Hayat MJ, Howlader N, Reichman ME, Edwards BK. Cancer statistics, trends, and multiple primary cancer analyses from the Surveillance, Epidemiology, and End Results (SEER) Program. The oncologist. 2007;12(1):20-37.

32. Patel AV, Hildebrand JS, Gapstur SM. Body mass index and all-cause mortality in a large prospective cohort of white and black U.S. Adults. PLoS One. 2014;9(10):e109153. Epub 2014/10/09. doi: 10.1371/journal.pone.0109153. PubMed PMID: 25295620; PubMed Central PMCID: PMCPMC4189918.

33. INEGI. Encuesta Nacional de Ingresos y Gastos de los Hogares 2020 (ENIGH). Mexico City, MX: 2021.

34. Quality AfHRa. Medical Expenditure Panel Survey In: Quality AfHRa, editor.: Agency for Healthcare Research and Quality; 2012.

35. Rodriguez Bolanos Rde L, Reynales Shigematsu LM, Jimenez Ruiz JA, Juarez Marquezy SA, Hernandez Avila M. [Direct costs of medical care for patients with type 2 diabetes mellitus in Mexico micro-costing analysis]. Rev Panam Salud Publica. 2010;28(6):412-20. Epub 2011/02/11. doi: 10.1590/s1020-49892010001200002. PubMed PMID: 21308167.

36. Villarreal-Rios E, Mathew-Quiroz A, Garza-Elizondo ME, Nunez-Rocha G, Salinas-Martinez AM, Gallegos-Handal M. [Cost of care of arterial hypertension and its impact on the budget assigned to health in Mexico]. Salud Publica Mex. 2002;44(1):7-13. Epub 2002/03/26. PubMed PMID: 11910723.

37. Gordois A, Scuffham P, Shearer A, Oglesby A. The health care costs of diabetic nephropathy in the United States and the United Kingdom. Journal of diabetes and its complications. 2004;18(1):18-26.

38. Barcelo A, Aedo C, Rajpathak S, Robles S. The cost of diabetes in Latin America and the Caribbean. Bull World Health Organ. 2003;81(1):19-27. Epub 2003/03/18. PubMed PMID: 12640472; PubMed Central PMCID: PMCPMC2572319.

39. Quintana-Carrillo RH, Arauz A, San-Juan D, Lopez de Santiago IT, Aguirre-Cruz L, Corona T, et al. The economic impact of smoking on the treatment costs of stroke at a tertiary care neurological center in Mexico. Int J Stroke. 2013;8(5):E23. Epub 2013/06/21. doi: 10.1111/ijs.12121. PubMed PMID: 23782735.

40. Prieto I, Bhattacharyya SK, Divino JC, Paniagua R, editors. Costs of Treating ESRD Patients With Various Dialysis Therapies in Mexico. iHEA 2007 6th World Congress: Explorations in Health Economics Paper; 2007.

41. Quintana M, Toriz JA, Novick D, Jones K, Botello BS, Silva JA. Resources and Costs Associated with the Treatment of Advanced and Metastatic Gastric Cancer in the Mexican Public Sector: A Patient Chart Review. Pharmacoecon Open. 2018;2(2):191-201. Epub 2018/04/07. doi: 10.1007/s41669-017-0043-2. PubMed PMID: 29623621; PubMed Central PMCID: PMCPMC5972114.

42. Meneses-Garcia A, Ramirez T, Ruiz-Godoy L, Chiquete E, Mohar A. Costs of breast cancer treatment prior to the introduction of immune-based therapy in Mexico. Rev Med Inst Mex Seguro Soc. 2012;50(1):19-24. Epub 2012/07/10. PubMed PMID: 22768813.

43. Gonzalez-Robledo MC, Wong R, Ornelas HA, Knaul FM. Costs of breast cancer care in Mexico: analysis of two insurance coverage scenarios. Ecancermedicalscience. 2015;9:587. Epub 2015/11/12. doi: 10.3332/ecancer.2015.587. PubMed PMID: 26557885; PubMed Central PMCID: PMCPMC4631579.

44. Granados-Garcia V, Pina-Sanchez P, Reynoso-Noveron N, Flores YN, Toledano-Toledano F, Estrada-Gomez G, et al. Medical Cost to Treat Cervical Cancer Patients at a Social Security Third Level Oncology Hospital in Mexico City. Asian Pac J Cancer Prev. 2019;20(5):1547-54. Epub 2019/05/28. doi: 10.31557/APJCP.2019.20.5.1547. PubMed PMID: 31128061; PubMed Central PMCID: PMCPMC6857888.

45. Yang D, Li T, Liu Z, Arbez N, Yan J, Moran TH, et al. LRRK2 kinase activity mediates toxic interactions between genetic mutation and oxidative stress in a Drosophila model: suppression by curcumin. Neurobiology of disease. 2012;47(3):385-92. doi: 10.1016/j.nbd.2012.05.020. PubMed PMID: 22668778.

46. Institute for Health Metrics and Evaluation (IHME). Global Burden of Disease Study 2019 (GBD 2019) Disability Weights. In: Global Burden of Disease Collaborative Network, editor. Seattle, Washington 2020.
